# Liquiritigenin Inhibits Tumor Growth and Vascularization in a Mouse Model of Hela Cells

**DOI:** 10.3390/molecules17067206

**Published:** 2012-06-12

**Authors:** Yuxin Liu, Sirou Xie, Yu Wang, Kang Luo, Yang Wang, Yunqing Cai

**Affiliations:** Department of Nutrition and Food Hygiene, School of Public Health, Nanjing Medical University, 140 Hanzhong Road, Nanjing 210029, Jiangsu, China

**Keywords:** cervical cancer, liquirtigenin, angiogenesis, vascular endothelial growth factor (VEGF)

## Abstract

Angiogenesis is one of the crucial steps in the transition of a tumor from a small, harmless cluster of mutated cells to a large, malignant growth, capable of spreading to other organs throughout the body. Vascular endothelial growth factor (VEGF) that stimulates vasculogenesis and angiogenesis is thought to be as an anti-angiogenic target for cancer therapy. Liquiritigenin (LQ), a flavanone existing in *Radix glycyrrhiza*, shows extensive biological activities, such as anti-inflammatory and anti-cancer properties. In our studies, liquiritigenin effectively inhibited the growth of tumors xenografted in nude mice from human cervical cancer cell line HeLa cells, and microvascular density (MVD) of the tumor exposed to liquiritigenin was reduced in a dose dependent manner, especially in the high dose group. Moreover, the expression and secretion of VEGF were down-regulated by the drug *in vivo* and *in vitro*. Therefore, liquiritigenin can be further studied on cancer and other diseases associated with VEGF up-regulation.

## 1. Introduction

In many malignancies, there are no useful therapeutic options. Cancer be detected only after a tumor mass undergoes continuous expansion [1]. However, expansion of a tumor mass beyond the initial physical constraint set by simple diffusion of nutrients and oxygen is dependent on angiogenesis [2,3]. Angiogenesis is a prerequisite for solid tumor growth. In addition, angiogenesis and vascularization assists tumor cells escape to the circulation and lodge in other organs [1,4]. However, angiogenesis, that is the formation of new blood vessels supporting tumor growth, is associated with increased risk of tumor invasion, metastasis, and patient mortality [5,6]. Targeting angiogenesis for cancer therapy remains an exciting area of preclinical and clinical studies. Vascular endothelial growth factor (VEGF), a key regulator of angiogenesis, directly stimulates endothelial cell proliferation and migration [7]. VEGF up-regulates angiogenesis stimulators. Inhibition of VEGF has shown efficacy in the treatment of several cancers, including cervical cancer and colorectal cancer. Many agents targeting VEGF have been developed, but most of the current anti-VEGF agents have some side effects such as hypertension, bleeding, gastrointestinal perforation, etc. if used chronically [8]. Bevacizumab (Avastin) working as a humanized monoclonal antibody, which prevented the formation of new blood vessels by targeting and inhibiting the function of VEGF, was approved by the U.S. Food and Drug Administration (FDA) for cancers in 2004 [9].

While many cancer therapies fall short of their anticipated clinical benefits and cause many adverse reactions, the diet-based anti-angiogenesis approach is being actively researched, as it has advantages to ensure the safety of medicines in human use. Flavanoids show potential therapy for cancer treatment and preventing recurrence, providing lower disease toxicity and increasing survival time [10]. Several studies on flavonoids showed that it inhibits the proliferation of cancer cells by blocking tumor-induced neovascularization through inhibition of the VEGF-induced formation of capillary-like structures, decreasing the levels of VEGF [11]. Among the constituents in licorice, liquiritigenin (7,4'-dihydroxyflavanone, LQ) is a member of the flavonoids, and one of the components of traditional Chinese medicines and foods, which have been shown to possess antioxidant, anti-inflammatory and anticancer properties [12,13].

Many different flavonoids have been used to change the systemic levels of angiogenic switches in order to inhibit angiogenesis and counteract cancer growth. Our previous studies have shown that the cytotoxicity of liquiritigenin on SMMC-7721 cells may occur via its effect on the generation of ROS, leading to cell apoptosis [14]. Furthermore, liquiritigenin induces apoptosis in part via the mitochondrial pathway, which is associated with p53 up-regulation, release of cytochrome c and elevated activity of caspase-9 and -3 in HeLa cells [15]. *In vivo*, liquiritigenin significantly inhibits hepatoma 22 tumors [16] The present study provided valuable insights into the mechanism of the anti-angiogenic and anti-migration effect of liquiritigenin *in vitro* [17,18]. Thus, we were able to identify a possible mechanism for the antitumor effect of LQ, involving liquirtigenin inhibition of tumor growth and vascularization *in vivo*.

## 2. Results and Discussion

### 2.1. Liquiritigenin Attenuate the Rate of Tumor Growth *in Vivo*

**Figure 1:**
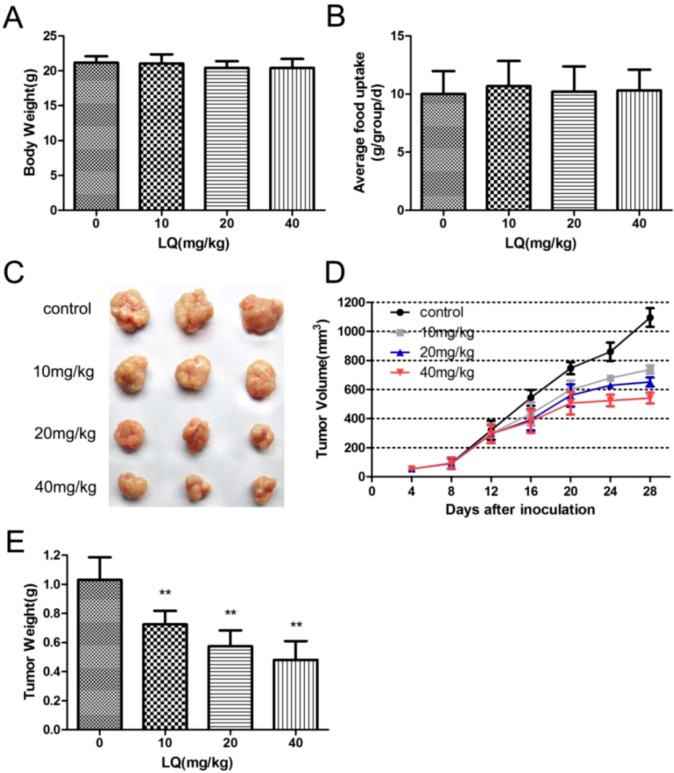
LQ inhibited xenograft of HeLa human cervical cancer cells in nude BALB/c mice. Nude BALB/c mice were inoculated with 2 × 10^6^ HeLa cells and treated LQ at a dose of 10, 20, 40 mg/kg respectively once per day for four weeks. Six mice were used per group. Tumor sizes were measured with a caliper and expressed as (r^2^ × l) / 2. At day 28, mice were sacrificed and tumor tissues were removed, photographed (**C**), and weighed (**F**). (**A**) body weights *in vivo* after treatment. Data are plotted as average body weights (g) per site versus group ± SD. (**B**) food uptake were measure every three days until the experiments were terminated. Data are plotted as average food weights (g) *per* site *versus* group ± SD.(**C**) Representative photographs of tumor sizes. (**D**) Time-related changes in solid tumor volumes. Tumors were measured daily by calipers starting at first appearance (day 4) and continued for 28 days. Data are plotted as tumor volume (mm^3^) *per* site *vs.* time (days) ± SD. (**E**) Tumor wet weight. At sacrifice all tumors were removed and weighed. Data are plotted as average tumor mass (g) per site versus group ± SD. ** *p* < 0.01, compared with control group.

The human xenograft tumor model in mice has been utilized for efficacy studies [19]. Treatments were initiated when the size of the tumor was approximately 0.03–0.05 cm^3^. During drug treatments, two peptides inhibited the growth of tumors originating from human epithelial cell line HeLa cells (Figure 1C) As shown in Figure 1F, tumors inhibitory rates of liquiritigenin at high-dosage had risen to more than 50%.

**Figure 2:**
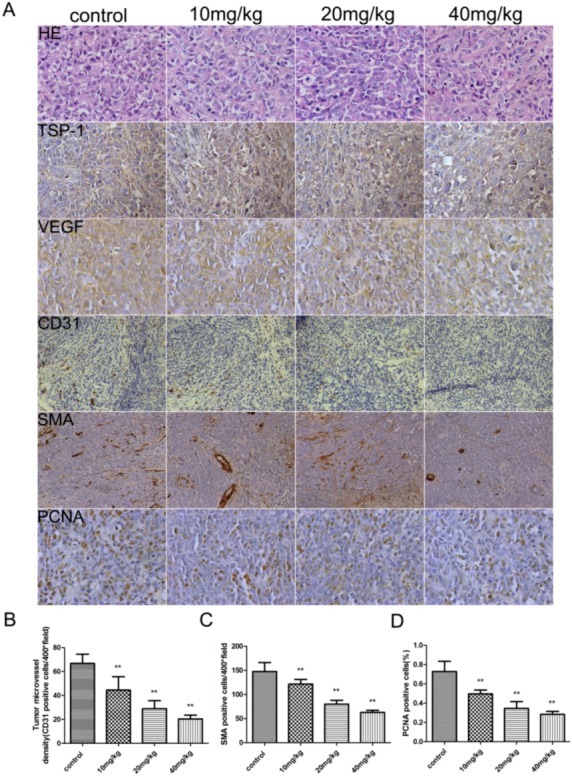
Immunohistochemical analysis of the effects of LQ on the angiogenesis of xenograft tumor. HeLa tumors were analyzed by hematoxylin-eosin staining and immunohistochemical staining for the expression of TSP-1,VEGF, CD31, SMA and PCNA (**A**). Microvessel density of the tumor tissues was assessed by CD31 and SMA immunohistochemical analysis (**B**). Anti-PCNA staining in tumor sections from each group, the percentage of PCNA-positive cells per 200 × field was accounted for as described in the methods (**C**). Data are mean ± SD. ** *p* <0.01, *vs.* control.

To evaluate whether the suppression of tumor growth is associated with angiogenesis, we examined the expression of endothelial marker CD31and α-SMA, VEGF, TSP-1 and Proliferating Cell Nuclear Antigen (PCNA) in HeLa tumors by immunostaining (Figure 2A).

The expression of VEGF in tumors was suppressed in a dose-dependent manner, but did not affect TSP-1 expression. Microvascular density (MVD) was determined by counting the number of the microvessels per high-power field (hpf) in a section with an antibody reactive to CD31 and α-SMA (Figure 2B,C).The mean MVD was reduced in tumors treated with drug, which showed a significant positive correlation with tumor size. A marked decrease of PCNA-positive cells was observed in the groups treated with liquiritigenin (Figure 2D). It was indicated that liquiritigenin could suppress the proliferation of tumor cells by inhibiting VEGF expression to prevent the angiogenesis of tumors.

### 2.2. Liquiritigenin Reduced the Expression of VEGF in HeLa Cells

**Figure 3:**
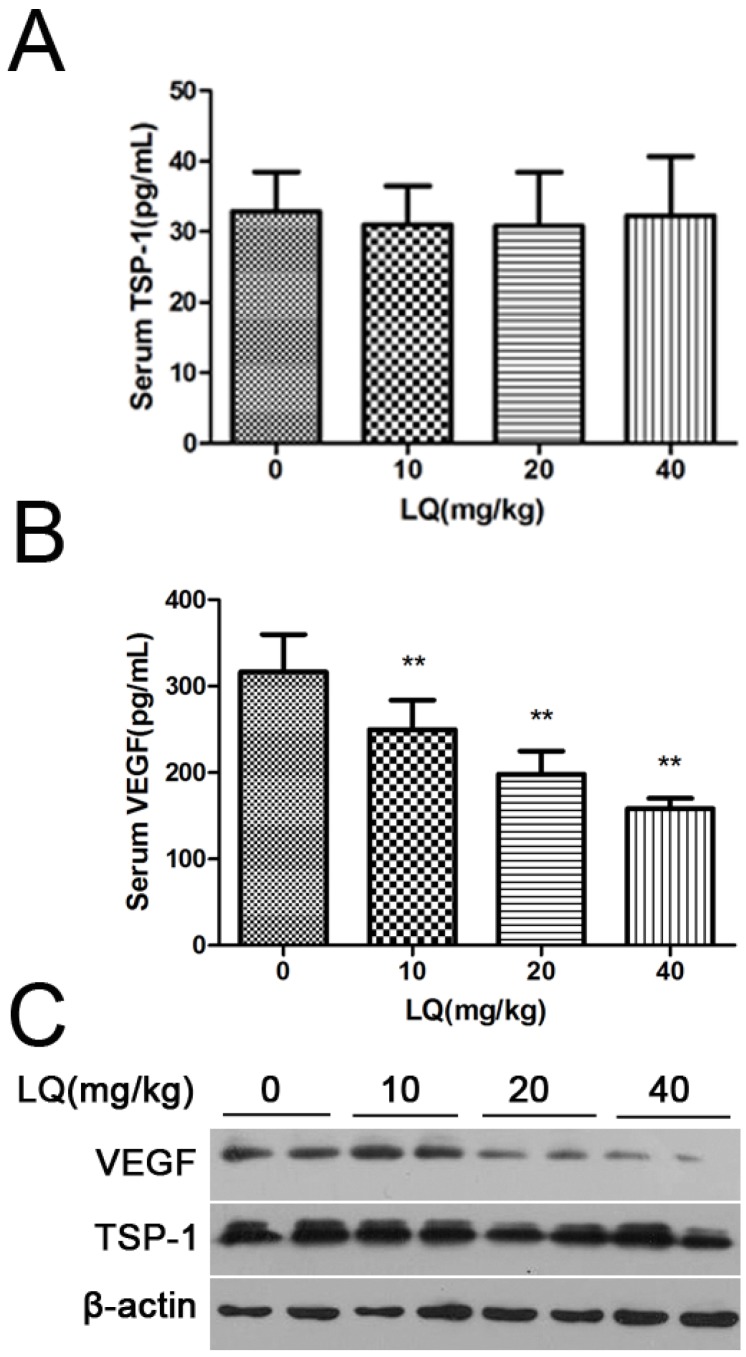
The effect of LQ on the expression and secretion of VEGF in Hela tumors. Serum thrombospondin-1 and VEGF concentrations were measured with ELISA kits, respectively (**A.B**.). Western blotting of tumoral VEGF protein at the different doses of tumor. In mice bearing tumors, treatment with LQ let to dose-dependent suppression of tumoral VEGF levels. Values are the means ± SD (n = 3) of three individual experiments. ** *p* < 0.01 *vs.* control.

To reinforce the physiological relevance of this observation *in vivo*, we investigated the effect of liquiritigenin on the key regulators involved in angiogenesis. Vascular endothelial growth factor (VEGF) plays a key role in physiological blood vessel formation and pathological angiogenesis such as tumor growth and ischemic diseases [20]. The secreted VEGF is a major angiogenic factor that regulates multiple endothelial cell functions, including mitogenesis. Cellular and circulating levels of VEGF are elevated in cervical cancer and are adversely associated with prognosis. Our results shown that liquiritigenin could down-regulate the expression of VEGF to reduce the level of secreted VEGF (Figure 3). And it has been reported that thrombospondin-1 (TSP-1) can inhibit angiogenic responses directly by interacting with VEGF and indirectly by engaging several endothelial cell TSP-1 receptors [21], so we were disappointed to see that liquiritigenin had no regulation effects on TSP-1 (Figure 3). Therefore, liquiritigenin could inhibit the angiogenesis of tumors by regulating VEGF expression.

### 2.3. Discussion

Liquiritigenin (LQ) is a flavanone extracted from Glycyrrhizae, and it has multiple biological effects, such as anti-inflammation and anti-cancer. A previous report indicated that liquiritigenin inhibited serum-induced HIF-1α and VEGF Expression via the AKT/mTOR-p70S6K signaling pathway in HeLa cells [17], but the anti-cancer effect of liquiritigenin *in vivo* remains unclear. In our study, liquiritigenin inhibited the growth of tumors in nude mice originating from human cervical cancer cell line HeLa cells, and reduced angiogenesis in a dose dependent manner (Figure 2). Therefore, the effect of liquiritigenin on tumor growth was potentially associated with inhibition of VEGF-mediated tumorigenesis and metastasis.

Cervical cancer is the term for a malignant neoplasm arising from cells originating in the cervix uteri. One of the most common symptoms of cervical cancer is abnormal vaginal bleeding, but in some cases there may be no obvious symptoms until the cancer has progressed to an advanced stage. Cervical cancer is almost always caused by human papillomavirus (HPV) infections [22]. Worldwide, cervical cancer is second most common and the fifth most deadly cancer in women [23]. The major treatments include surgery, chemotherapy, radiation therapy, immunotherapy, and vaccine therapy. Recently, combination neoadjuvant chemotherapy with paclitaxel and cisplatin has been shown to potentially improve long-term survival of patients with uterine cervical cancer [24]. However, these molecules display serious side effects in patients treated for a long time. Thus, the research on drug design and discovery offering various advantages, such as high specificity and low toxicity, is one of the most promising fields in the development of new drugs.

VEGF plays an important role in the regulation of tumor growth and metastasis, of which expression is correlated with disease behaviors in various cancers, especially for cervical cancer [25]. Elevated pre-therapeutic serum VEGF levels are associated with poor responses and a shorter time to progression in patients with cervical cancer undergoing primary radiotherapy [26]. Therefore, VEGF has been considered as a potential therapeutic target for cervical cancer and other aggressive malignant tumors.

In our previous study, liquiritigenin had the strongest effect on inhibition of the expression and release of VEGF in HeLa cells among drug candidates for our studies. and many reports have shown that there are potential anticancer action of plant flavonoids [27]. In order to confirm the inhibiting effects of liquiritigenin on cancer cellular growth *in vivo*, a human xenograft tumor model in nude mice was utilized for efficacy studies [28]. During the treatment with liquiritigenin, the growth of tumors originating from human cervical cancer cell line HeLa cells was inhibited (Figure 1). Angiogenesis is required for tumor growth and metastasis and constitutes an important point in the control of cancer progression [29,30]. The VEGF expression in tumors was observed by immunostaining, and liquiritigenin could suppress the expression of VEGF in a dose dependent manner. It was observed that there was a significant differences of the endothelial area marked with CD31 and α-SMA in between treatment group and control group (Figure 2). It has been reported that the imbalance between circulating concentrations of the angiogenesis inhibitor TSP-1 and the activator VEGF played an important role in tumor progression [21,31,32]. As shown in Figure 2 and Figure 3, liquiritigenin had no effect on TSP-1 expression, which suggested that liquiritigenin could inhibit the expression of VEGF by regulation of the upstream signaling of VEGF. Overall, this study describes the discovery of a flavanone, liquiritigenin, which is an effective inhibitor of VEGF. This molecule may have further utility in clinical applications for treating cancer and other diseases associated with VEGF up-regulation.

## 3. Experimental

### 3.1. Chemicals and Drugs

LQ (purity ≥99.68%) was purchased from Nanjing University of Chinese Medicine (Nanjing, China). It was suspended in 5% sodium carboxymethycellulose solution before use. Other chemicals were of the highest grade available. Cell culture reagents were obtained from Gibco Life Technology (Grand Island, NY, USA).

### 3.2. Cell Culture

HeLa cells were obtained as a gift from the Institute of Toxicology (Nanjing Medical University, China). Cells were maintained in Dulbecco’s modified Eagle’s medium (DMEM) supplemented with 10% heat-inactivated fetal bovine serum (FBS), 100 U/mL penicillin and 100 U/mL streptomycin at 37 °C in a humidified atmosphere with 5% CO_2_.

### 3.3. Mice and Tumor Xenografts

Five-week-old nude BALB/c mice and their diet were all obtained from Shanghai SLAC Laboratory Animal Co. Ltd (Shanghai, China). Experiments commenced after seven days of acclimatization. The mice were allowed *ad libitum* access to food and water. Nude BALB/c mice were used for subcutaneous implantation of human cervical tumor cell HeLa (2 × 10^6^ per site). Tumor-bearing mice were randomly divided into four groups with six mice *per* group. Three LQ groups with low, middle high doses (10, 20, 40 mg/kg, respectively) were administered intragastrically once per day starting on day 3 after innoculation. The control group was treated with the same volume of 5% sodium carboxymethycellulose. Mice were sacrificed after 28 days. All animal experiments were conducted in accordance with the Bioethics Committee guidelines in Nanjing Medical University, China. General reactions were observed every day after drug administration; mouse weights were measured by balance, respectively, every day and their food weight were measured every three days until the experiments were terminated. Tumor growth was measured with a microcaliper every four days throughout the experiment, Tumor volume was calculated as the following: volume = A·B 2/2, where A is the longer and B is the shorter diameter (mm).

### 3.4. Histopathological Evaluation

Tumor tissue was fixed in 4% paraformaldehyde solution for 48 h. The tissue pieces were dehydrated in increasing concentrations of ethanol, cleared in xylene, embedded in paraffin, and cut into 5-μm sections. After staining with hematoxylin and eosin (HE) routinely, the histological characteristics of the tumor tissues were observed and photographed under a microscope (Olympus, IX-70, Tokyo, Japan).

### 3.5. Immunohistochemistry

Paraffin sections were permeabilized with 36 μg/mL proteinase K (Roche Diagnostics Corp., Indianapolis, IN, USA) and stained with antibody against CD31 (platelet-derived endothelial cell adhesion molecule), SMA (smooth muscle actin), PCNA (proliferating cell nuclear antigen), VEGF (vascular endothelial growth factor), TSP-1 (PECAM) (BD Bioscience, Franklin Lakes, NJ, USA). Sections were photographed at ×200 magnification using an Olympus AX70 microscope (Melville, NY, USA). To evaluate tumor vessel area and microvessel density, tumor sections immunostained for CD31 were imaged at a magnification of ×200, and microvessel density and blood vessel area were counting the number of CD31-positive vessels and SMA-positive vessels in five objective fields of representative areas in each section.

### 3.6. Elisa Measurements

Their blood was collected from the orbital cavity vein plexus, and centrifuged to obtain serum. The expression of VEGF was determined using sandwich enzyme-linked immunosorbent assays (R&D Systems, Inc, Minneapolis, MN, USA) according to the manufacturer’s instructions. The expression of TSP-1 was determined using enzyme-linked immunosorbent assays (R&D Systems Europe Ltd, London, UK; ^#^DTSP10) according to the manufacturer’s instructions. Briefly, serum samples were diluted 1:100 and added to the precoated wells. Unbound substrate was washed away, and an enzyme-linked polyclonal antibody specific for VEGF and TSP-1 was added to the wells followed by subsequent washes and addition of color substrate. Individual well absorbance was measured at 450 nm using an EL 800 Universal Microplate Reader (Bio-Tek Instruments, Winooski, VT, USA).

### 3.7. Western Blot Analysis

SDS-/PAGE and Western blot analysis was performed on the 10,000 g supernatant fraction of total soluble proteins prepared from frozen tumor tissue (1,000 μg). Briefly, frozen tumor tissue was weighed and diced into small pieces. Ice cold lysis buffer (20 mM Tris HCl pH 7.4 with 150 mM NaCl, 1 mM EDTA, 0.5% NP-40) 0.5% sodium deoxycholate, 1 mM DTT, protease inhibitor cocktail (Roche), 1.5 mM sodium vanadate, 1 μM cystine and 80 mM sodium glycerophosphate was added, homogenized, and centrifuged at 10,000 g for 15 min. 10 μg protein was loaded in each lane on a 12% Bis-Tris gel, the protein transferred to a PVDF transfer membrane (Immobilon-P, Millipore, Billerica, MA, USA), and blocked overnight in 5% dry fat-free milk in TBST, and incubated (2 h) with indicated primary anti-bodies (VEGF, 1:200), followed by incubation (1 h) with secondary anti body, goat anti-mouse secondary antibody (1:16,000) (Sigma-Aldrich, St Louis, MO, USA); Immunoreactivity was visualised by enhanced chemiluminescence reagent (Perkin Elmer Cetus, Foster City, CA, USA). To demonstrate equal loading, blots were stripped and reprobed with a specific antibody recognizing β-actin (1:5,000 dilution; Sigma-Aldrich).

### 3.8. Statistical Analysis

All statistical analysis were conducted using One-way ANOVA with Dunnett’s post test using GraphPad InStat version 3.0 for Windows (GraphPad Software, San Diego, CA, USA).

## 4. Conclusions

Taken together, for the first time, our results demonstrate that LQ inhibited the tumor growth (HeLa cells inoculated in nude mice) *in vivo*. This study further observed that LQ markedly decreased MVD in tumors. We conclude that LQ-induced down-regulation of VEGF is part of a mechanism, which in turn leads to reduced angiogenesis, that could support LQ’s potential efficacy in clinical applications.
